# Workplace bullying and risk of suicide and suicide attempts: A register-based prospective cohort study of 98 330 participants in Denmark

**DOI:** 10.5271/sjweh.4034

**Published:** 2022-08-31

**Authors:** Paul Maurice Conway, Annette Erlangsen, Matias Brødsgaard Grynderup, Thomas Clausen, Reiner Rugulies, Jakob Bue Bjorner, Hermann Burr, Laura Francioli, Anne Helene Garde, Åse Marie Hansen, Linda Magnusson Hanson, Jonas Kirchheiner-Rasmussen, Tage S Kristensen, Eva Gemzøe Mikkelsen, Elsebeth Stenager, Sannie Vester Thorsen, Ebbe Villadsen, Annie Høgh

**Affiliations:** 1 Department of Psychology, University of Copenhagen, Denmark; 2 Danish Research Institute for Suicide Prevention, Mental Health Centre Copenhagen, Capital Region of Denmark, Copenhagen, Denmark; 3 Copenhagen Research Centre for Mental Health, Capital Region of Denmark, Copenhagen, Denmark; 4 Department of Mental Health, Johns Hopkins Bloomberg School of Public Health, Baltimore, MD, United States; 5 Centre for Mental Health Research, Australian National University, Canberra, ACT, Australia; 6 National Research Centre for the Working Environment, Copenhagen, Denmark; 7 Department of Public Health, University of Copenhagen, Copenhagen, Denmark; 8 Optum Patient Insights, Lincoln, RI, USA; 9 Department of Work and Health, Federal Institute for Occupational Safety and Health BAuA, Berlin, Germany; 10 Stress Research Institute, Stockholm University, Stockholm, Sweden; 11 Task-Consult, Gilleleje, Denmark; 12 Department of Psychology, University of Southern Denmark, Odense, Denmark; 13 Focused Research Unit in Psychiatry, Institute of Regional Health Research, University of Southern Denmark, Odense, Denmark

**Keywords:** offensive behavior, death by suicide, depression, harassment, mental health, register-based study, suicidal behavior

## Abstract

**Objectives:**

The aim of this study was to analyze whether individuals reporting exposure to workplace bullying had a higher risk of suicidal behavior, including both suicide attempt and death by suicide, than those not reporting such exposure.

**Methods:**

Using a prospective cohort study design, we linked data from nine Danish questionnaire-based surveys (2004–2014) to national registers up to 31 December 2016. Exposure to workplace bullying was measured by a single item. Suicide attempts were identified in hospital registers and death by suicide in the Cause of Death Register. Among participants with no previous suicide attempts, we estimated hazard ratios (HR) and 95% confidence intervals (CI), adjusting for sex, age, marital status, socioeconomic status, and history of psychiatric morbidity.

**Results:**

The sample consisted of 98 330 participants (713 798 person-years), 63.6% were women, and the mean age was 44.5 years. Of these participants, 10 259 (10.4%) reported workplace bullying. During a mean follow-up of 7.3 years, we observed 184 cases of suicidal behavior, including 145 suicide attempts, 35 deaths by suicide and 4 cases that died by suicide after surviving a suicide attempt. The fully-adjusted HR for the association between workplace bullying and suicidal behavior was 1.65 (95% CI 1.06–2.58). The HR for suicide attempts and death by suicide were 1.65 (1.09–2.50) and 2.08 (0.82–5.27), respectively. Analyses stratified by sex showed a statistically significant association between workplace bullying and suicidal behavior among men but not women.

**Conclusions:**

The results suggest that exposure to workplace bullying is associated with an elevated risk of suicidal behavior among men.

Globally, more than 817 000 deaths by suicide occur annually, while the number of persons with a non-fatal episode (ie, self-harm) might be 20 times higher (1). The suicide rate has declined globally over recent decades (2). In Denmark, the suicide rate has remained relatively stable in recent years, suggesting that new venues for intervention are needed.

Suicidal behavior has a complex etiology, with multiple contributions from biological, psychological, clinical, social, and environmental factors (3). Among social factors, repeated exposure to psychosocial work stressors may play a role in suicidality (4). Workplace bullying is an especially severe form of psychosocial stressor, involving a long-lasting exposure to repeated negative behaviors at work, such as harassment, offending or ostracizing individuals, or taking actions that negatively affect an individual’s job (5). The average prevalence of workplace bullying worldwide is estimated as 14.6%, ranging from 11.3% – when workplace bullying is measured using the self-labelling method (as it is the case in the present study) – to 18.1%, when workplace bullying is instead measured using the behavioral experience approach (6). The available evidence supports a role of workplace bullying in the onset of mental disorders, especially depression (7, 8), and suicidal ideation (9, 10), which are key antecedents of suicidal behavior (11). A link between workplace bullying and both mental disorders and suicidality appears plausible, considering that exposure to workplace bullying may cause severe psychological pain, including feelings of hopelessness, entrapment, loss of control, worthlessness, social exclusion, deterioration of self-esteem, and chronic psychological distress (12). However, prospective studies on the association between workplace bullying and subsequent risk of suicidal behavior, including suicide attempt and death by suicide, are lacking, with the only available evidence supporting a prospective association between workplace bullying and suicidal ideation (13). We aim to fill this gap by examining, in a large Danish study linking pooled survey data to national register data, the prospective association between exposure to workplace bullying and subsequent suicidal behavior, including suicide attempts and death by suicide. To avoid selective reporting and other post-hoc decision-making biases, a protocol detailing the analytical plan was published prior to the present study (14).

## Methods

### Study design and participants

We adopted a prospective cohort study design. At the Danish National Research Centre for the Working Environment, we created a single dataset by pooling together questionnaire data collected from 2004–2014 in nine Danish surveys, all of which contained an item on self-reported workplace bullying. The surveys included individuals employed in different occupational groups in both the private and public sector (see supplementary material www.sjweh.fi/article/4034, table S1, for details about the surveys included). The nine surveys provided 14 waves of measurement in all, since four of the surveys [The Danish Work Environment Cohort Study (DWECS), Workplace Bullying and Harassment (WBH), Social and Health Care Study (SOSU), and Work Environment and Health (WEHD)] comprised more than one wave. Using the unique personal identifier, assigned to all Danish residents (15), the pooled dataset was linked to the following national registers: the Danish Civil Register (since 2004) (16), the Danish National Patient Register (since 1977) (17), the Danish Psychiatric Central Research Register (since 1994) (18), the Danish Register of Causes of Death (since 2004) (19), and the Income Statistics Register (since 2004).

Those individuals who responded to the item on self-reported workplace bullying in these surveys were included in the present study. With regard to the four surveys with more than one wave, the first wave in which participants provided a valid response to the item on workplace bullying was used to determine exposure status (ie, exposed or non-exposed to workplace bullying). For instance, if in the three-wave SOSU cohort, a participant provided a valid answer to the item of workplace bullying in the second but not the first wave, the second wave was used to classify the participant as exposed or not exposed. The date the question on workplace bullying was answered was considered as the date of exposure and follow-up started on the following day. If the response date was missing, date of exposure was considered as the date the survey questionnaire was sent out. With regard to the study outcomes (suicide attempt and death by suicide), participants were followed in the registers from the date they completed the questionnaire survey until 31 December 2016. Participants, who migrated or died by causes other than suicide, were censored from the study at the time of the respective event.

**Figure 1:**
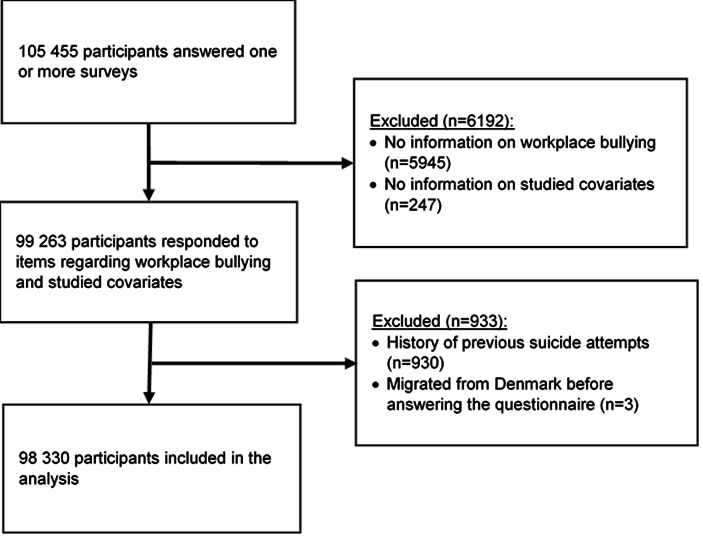
Participants’ inclusion criteria.

Overall, the pooled dataset included 139 575 questionnaire responses, corresponding to 105 455 unique participants. First, we excluded 6192 (5.9%) participants with missing data on workplace bullying (N=5945) and the covariates sex, age, marital status, and socio­economic status (N=247), resulting in 99 263 participants (94.1%). Next, we excluded 930 (0.9%) participants with previous suicide attempts. We finally excluded three participants who were officially listed as having migrated out of the country on the date when they answered the questionnaire. The application of these further exclusion criteria resulted in a final sample of 98 330 (93.2%) participants included in the present analyses (figure 1). A comparison of the sociodemographic characteristics between participants in the final sample and participants who had been excluded due to missing values is provided in supplementary table S3.

### Ethics committee approval

The study followed the principles outlined in the Declaration of Helsinki. The Danish Data Protection Agency approved the project (Capital Region of Denmark; j.nr.: 2012-58-0004) and data are stored on a secure server at Statistics Denmark. According to Danish law, studies that use questionnaire and register data only do not require approval from the National Committee on Health Research Ethics.

### Workplace bullying

In seven of the nine surveys, self-reported exposure to workplace bullying was measured retrospectively with the following questionnaire item: “Have you been subjected to bullying at work within the past 12 months?”, to be answered using a Likert-type scale with five response options; 1=never, 2=now and then, 3=monthly, 4=weekly, and 5=daily. In the Nursing Work Environment, Well-being and Health (SATH), DWECS 2005, and DWECS 2010 surveys, exposure frequency was measured with a dichotomous item (1=no, 2=yes). In WBH 2006 and WBH 2008, the format with five response options was used but the retrospective exposure time was 6 instead of 12 months. For all the surveys included, the questionnaire item was preceded by a definition of workplace bullying, which is useful to calibrate responses by reducing the potential influence of individual differences in the interpretation of the exposure. To harmonize measures across surveys, we created the following dichotomous exposure variable that was used in all the analyses of the present study: 1=0 “non-exposed to workplace bullying” (reference); 2–5=1 “exposed to workplace bullying”.

### Suicidal behavior

The primary outcome, suicidal behavior, was defined as a first episode of a suicide attempt or death by suicide during follow-up. Suicide attempts were identified through contacts to somatic or psychiatric hospitals in the Danish National Patient Register (17) and the Danish Psychiatric Central Research Register (18). This comprised persons who had been registered with a main or sub-diagnosis, according to the 10^th^ revision of the International Classification of Diseases (ICD), which indicated a suicide attempt (ICD-10: X60-X84) or that the reason for contact was a suicide attempt. All types of hospital contacts, ie, emergency department, inpatient, and outpatient, were included. Participants were classified as cases of suicide attempt at the first date of the event, and then censored (ie, repeated events of suicide attempt were not considered). Information on individuals who died by suicide was retrieved from the Danish Register of Causes of Death, using the ICD-10 codes X60-X84 (19). The date of death was considered as the date of the outcome. Participants were censored after the first episode of suicide behavior. Participants registered with a suicide attempt before the baseline date were excluded from the study.

### Covariates

The following covariates were included: sex (men, women); age (continuous variable); marital status (unmarried; married/cohabiting/registered partnership; divorced; widowed); socioeconomic status, coded based on Denmark Statistics’ classification (Low, Medium, High, Student/Other); history of mental disorders (yes/no; identified as ICD-8 codes 290-316 or ICD-10 codes F00-F99); history of a psychotropic drug prescription (yes/no; identified as prescription of antipsychotics [N05A], anxiolytics [N05B], anxiolytics, hypnotics and sedatives [N05C], antidepressants [N06A] or psychostimulants, agents used for ADHD and nootropics [N06B]).

Data on sex, age, and marital status were obtained from the Danish Civil Register (16), and information on socioeconomic status was derived from the Income Statistics Register. Information on history of mental disorders and psychotropic drug prescriptions was retrieved from the Danish Psychiatric Central Research Register and the Danish National Prescription Registry (20), respectively. Since most surveys asked about workplace bullying during the previous 12 months, information on history of mental disorders and psychotropic drug prescriptions were included from 1 January 2000 up to 12 months before survey participation.

### Statistical analyses

*Main analysis*. Using multivariable Cox proportional hazard models, hazard ratios (HR) and 95% confidence intervals (CI) were calculated to estimate the association between workplace bullying and subsequent suicidal behavior. Robust clusters based on the survey waves were used to account for intra-group correlations due to the clustering of participants in different surveys (21). The proportional hazards assumption was tested and confirmed by Schoenfeld’s residuals (P>0.05), and the visual inspection of the observed Kaplan-Meier survival curves and the log-log plots (data not shown).

Next to a crude analysis, we adjusted for sex, age, marital status and socioeconomic status (model 1). We additionally adjusted for history of mental disorders (model 2), considering that individuals with mental disorders might have an elevated risk of both being exposed to workplace bullying and engaging in suicidal behavior (11).

*Supplementary analyses*. First, we estimated the associations between workplace bullying and suicide attempt and death by suicide as separate outcomes. Second, we conducted sub-group analyses with respect to sex, age dichotomized (<31 versus ≥31 years), socioeconomic status and marital status, and calculated multiplicative interaction terms in the fully adjusted model. Third, we examined the association between workplace bullying and suicidal behavior while excluding four participants who had a record of both suicide attempt and death by suicide. Fourth, we examined the association between workplace bullying and suicidal behavior adjusting for history of psychotropic drug prescriptions, instead of history of mental disorders. Fifth, we calculated the association between workplace bullying and suicide behavior stratified by cases that occurred in the first four years of follow-up and cases that occurred later. Sixth, we calculated the association between the covariates and suicidal behavior.

All analyses were performed following the procedure described in the pre-published protocol (14), with the exception of the calculation of interaction terms, which was an authors’ post-hoc decision. Other analyses not described in the pre-published protocol (exclusion of the four participants who were recorded both with a suicide attempt and later with death by suicide and stratification of suicidal behavior by cases that occurred during the first four years of follow-up and cases that occurred later) were included during the revision process. All analyses were conducted using the statistical package STATA 16.1 (StataCorp LP, College Station, TX, USA).

## Results

The mean study follow-up time was 7.3 years [range 1 day to 12.1 years; median 8.1, standard deviation (SD) 3.3 years], yielding 713 798 person-years. The characteristics of the study sample are shown in table 1. Details for each of the 14 samples are provided in supplementary table S2. The sample consisted of 63.6% women, and the mean age was 44.5 years. Overall, 10 259 participants (10.4%) reported exposure to workplace bullying. During follow-up, 184 cases of suicidal behavior were identified, consisting of 145 suicide attempts, 35 death by suicide and 4 cases that first had a suicide attempt and later died of suicide. These 4 cases were included in both analyses on suicide attempt and death by suicide. The mean number of years and standard deviation between baseline and suicide events were 4.0 (SD 2.8) for suicidal behavior (N=184), 3.9 (SD 2.9) for suicide attempt (N=149), and 4.6 (SD 2.6) for death by suicide (N=39). Of all participants, 3.1% and 21.3% had a history of diagnosed mental disorders and a history of psychotropic drug prescriptions, respectively.

**Table 1:** Characteristics of study sample (N=98 330). [SD=standard deviation.]

	Total N (%)	Mean (SD)
Sample	98 330 (100)	
Age		44.5 (11.2)
Age (years) dichotomized		
<31	13 327 (13.6)	
≥31	85 003 (86.4)	
Women	62 582 (63.6)	
Participants reporting exposure to workplace bullying	10 259 (10.4)	
Marital status		
Living alone	12 332 (12.5)	
Cohabiting	78 568 (79.9)	
Divorced	6208 (6.3)	
Widow (no)	1222 (1.2)	
Socioeconomic status ^a^		
Low	37 595 (38.2)	
Medium	≈24 900 ^b^ (≈25)	
High	≈19 210 ^b^ (≈20)	
Student/Other	16 627 (16.9)	
Diagnoses of any mental disorder	3064 (3.1)	
Psychotropic drug prescriptions	20 901 (21.3)	

a For socioeconomic status: low=employed in a job requiring skills on a basic level; medium=employed in a job requiring skills on the mid-level; high=leaders, both employed and self-employed with subordinates, and participants employed in a job requiring skills on the highest level; student/ other=students and self-employed without subordinates.

b The exact total number of participants is not reported for high and medium socioeconomic status to avoid being able to calculate the numbers for SOSU U by subtraction (see supplementary table S2). The symbol ≈ indicates approximate numbers.

### Main analysis

**Figure 2:**
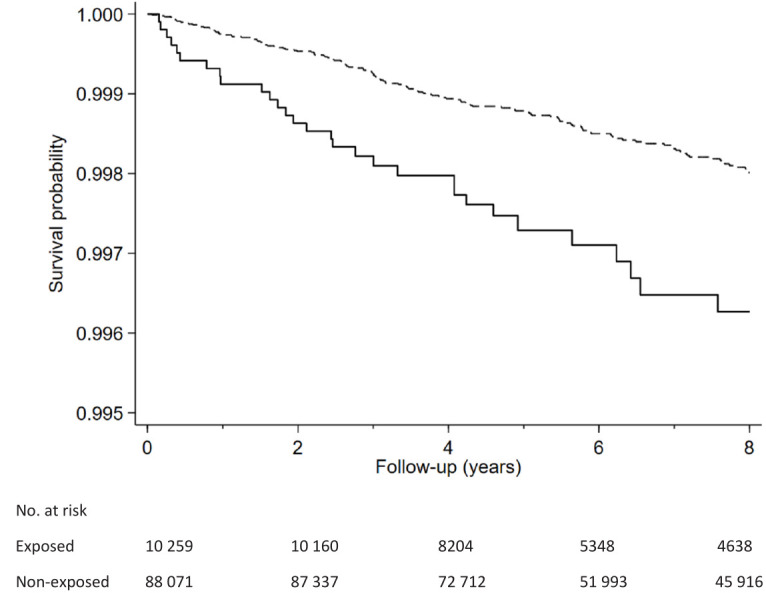
Kaplan-Meier curve for the probabilities of suicidal behavior for participants exposed (solid line) and not exposed (dashed line) to workplace bullying.

Figure 2 shows the Kaplan-Meier curves for the probabilities of suicidal behavior for participants exposed and not exposed to workplace bullying during follow-up. The curves indicate that the proportional hazard assumption was fulfilled and that exposed participants showed more suicidal behavior. The corresponding HR are presented in table 2. Participants exposed to workplace bullying at baseline had a statistically significant elevated risk of suicidal behavior compared with the non-exposed (HR 1.83, 95% CI 1.20–2.78; P=0.002) in the crude model. The association remained statistically significant after adjusting for sex, age, marital status, and socioeconomic status (HR 1.77, 95% CI 1.15–2.70; model 1), and additionally for previous history of diagnosed mental disorders (HR 1.65, 95% CI 1.06–2.58; model 2).

**Table 2:** Hazard ratios (HR) of suicidal behavior, suicide attempt and death by suicide. [WB=workplace bullying]

	N (%) of suicidal events ^a^	Person-years	Rate per 100 000 person-years	Crude HR (95% CI)	Model 1^b^ HR (95% CI)	Model 2^c^ HR (95% CI)
Suicidal behavior						
Non-exposed to WB	153 (83.2)	642 571	23. 81	1.00	1.00	1.00
Exposed to WB	31 (16.8)	71 227	43.52	1.83 (1.20–2.78)	1.77 (1.15–2.70)	1.65 (1.06–2.58)
Suicide attempt						
Non-exposed to WB	124 (83.2)	642 571	19.30	1.00	1.00	1.00
Exposed to WB	25 (16.8)	71 227	35.01	1.81 (1.22–2.70)	1.77 (1.19–2.63)	1.65 (1.09–2.50)
Death by suicide						
Non-exposed to WB	31 (79.5)	643 183	4.82	1.00	1.00	1.00
Exposed to WB	8 (20.5)	71 391	11.21	2.34 (0.98–5.62)	2.21 (0.87–5.64)	2.08 (0.82–5.27)

a Four participants first attempted suicide, followed by death by suicide during follow-up. These participants are included in all three analyses. For the analyses of suicide behavior and suicide attempt, case status is determined on the date of the suicide attempt; for death by suicide, case status is determined based on the date of death.

b Model 1 was adjusted for sex, age, marital status, and socio-economic status.

c Model 2 was adjusted for the same covariates as in Model 1 plus previous history of diagnosed mental disorders.

### Supplementary analyses

When we analyzed suicide attempt and death by suicide separately (table 2), exposure to workplace bullying was associated with both suicide attempts (fully-adjusted HR 1.65, 95% CI 1.09–2.50) and death by suicide (fully-adjusted HR 2.08, 95% CI 0.82–5.27), although the CI for death by suicide were wide and included unity. The Kaplan-Meier curves for suicide attempt and death by suicide are provided in supplementary figures S1 and S2.

Table 3 shows the analyses stratified by sex, age, socioeconomic status, and marital status. The HR for the association between workplace bullying and suicidal behavior were similar for younger (<31 years) versus older (≥31 years) participants (P for multiplicative interaction=0.969), for participants with low versus medium/high socioeconomic status (P for multiplicative interaction=0.458), and for persons living alone versus cohabiting persons (P for multiplicative interaction=0.216) and divorced (P for multiplicative interaction=0.860), with overlapping CI. With regard to sex, the HR in the fully-adjusted model were statistically significant among men (HR, 2.92; 95% CI 1.74–4.91) but not among women (HR, 1.12; 95% CI 0.71–1.78), with a P for multiplicative interaction of 0.002, indicating effect modification by sex.

**Table 3:** Hazard ratios (HR) of suicidal behavior stratified by sex, age dichotomised, socio-economic status and marital status. Results are not reported for widowed respondents due to too few cases of suicidal events. [WB=workplace bullying]

	N (%) of suicidal events	Person-years	Rate per 100 000 person-years	Crude HR (95% CI)	Model 1 HR (95% CI)	Model 2 ^e^ HR (95% CI)
Men (N=35 748)						
Non-exposed to WB	47 (74.6)	205 950	22.82	1.00	1.00	1.00
Exposed to WB	16 (25.4)	20 961	76.33	3.32 (2.04–5.40)	3.03 (1.81– 5.06) ^a^	2.92 (1.74–4.91)
Women (N=62 582)						
Non-exposed to WB	106 (87.6)	436 620	24.28	1.00	1.00	1.00
Exposed to WB	15 (12.4)	50 266	29.84	1.23 (0.79–1.92)	1.23 (0.79–1.91) ^a^	1.12 (0.71–1.78)
Aged <31 (N=13 327)						
Non-exposed to WB	28 (84.8)	89 017	31.45	1.00	1.00	1.00
Exposed to WB	5 (15.2)	8 530	58.62	1.84 (0.59–5.77)	1.62 (0.54–4.87) ^b^	1.55 (0.51–4.72)
Aged ≥31 (N=85 003)						
Non-exposed to WB	125 (82.8)	553 554	22.58	1.00	1.00	1.00
Exposed to WB	26 (17.2)	62 697	41.47	1.84 (1.17–2.90)	1.76(1.09–2.83) ^b^	1.64 (1.00–2.71)
Low/Student/Other socio-economic status ^f^ (N=37 595)						
Non-exposed to WB	75 (83.3)	259 438	28.91	1.00	1.00	1.00
Exposed to WB	15 (16.7)	32 067	46.78	1.61 (0.87–2.98)	1.62 (0.88–2.96) ^c^	1.53 (0.83–2.81)
Medium/High socio-economic statusf (N=44 108)						
Non-exposed to WB	43 (82.7)	279 417	15.39	1.00	1.00	1.00
Exposed to WB	9 (17.3)	27 105	33.20	2.17 (1.18–3.98)	2.03 (1.09–3.79) ^c^	1.87 (0.98–3.55)
Living alone (N=12 332)						
Non-exposed to WB	18 (75.0)	76 764	23.45	1.00	1.00	1.00
Exposed to WB	6 (25.0)	10 236	58.62	2.48 (1.26–4.86)	2.44 (1.24–4.81) ^d^	2.45 (1.25–4.82)
Cohabiting (N=78 586)						
Non-exposed to WB	120 (86.3)	518 842	23.13	1.00	1.00	1.00
Exposed to WB	19 (13.7)	53 221	35.70	1.72 (0.94–2.53)	1.51 (0.92–2.50) ^d^	1.38 (0.80–2.37)
Divorced (N=6208)						
Non-exposed to WB	13 (72.2)	38 896	33.42	1.00	1.00	1.00
Exposed to WB	5 (27.8)	6 754	74.03	2.23 (0.68 –7.27)	2.32 (0.72–7.56) ^d^	2.23 (0.69–7.21)

a Model 1 adjusted for age, marital status, and socio-economic status.

b Model 1 adjusted for sex, marital status, and socio-economic status.

c Model 1 adjusted for sex, age, and marital status.

d Model 1 adjusted for sex, age, and socio-economic status.

e Model 2 adjusted for the same covariates as in Model 1 plus previous history of diagnosed mental disorders.

f For the present analysis, socio-economic status was dichotomized into: Low/Student/Other and Medium/High.

When we excluded the four individuals who were recorded both with a suicide attempt and later with death by suicide, estimates were slightly attenuated but in the same direction as the estimates in the main analyses (supplementary table S4).

When we adjusted for history of psychotropic drug prescriptions, instead of history of diagnosed mental disorders, the fully-adjusted HR for suicidal behavior was comparable to the HR from the main analysis (supplementary table S5).

When we stratified suicidal behavior by cases that occurred during the first four years of follow-up and cases that occurred later, the associations were slightly stronger for cases occurring in the first four years (Supplementary tables S6 and S7).

Supplementary table S8 shows the crude and adjusted HR for the covariates. Risk of suicidal behavior was higher for men than women, younger than older individuals, individuals with a lower socioeconomic status, and individuals with a history of diagnosed mental disorders compared to those without such a history.

## Discussion

Pooling data from 14 survey waves, we were able to generate the largest study sample to date on the association between exposure to workplace bullying and suicidal behavior. To our knowledge, this is the first prospective study examining such an association. We found an increased risk of suicidal behavior, including both suicide attempts and death by suicide, among men who had previously reported exposure to workplace bullying and had no previous history of suicide attempts and diagnosed mental disorders. The association between workplace bullying and suicidal behavior was not statistically significant among women.

Our findings are in agreement with earlier studies reporting associations between workplace bullying and the onset of suicidal ideation (9, 10) and mental disorders (7). A recent study reported a prospective association between another type of offending behavior, workplace sexual harassment, and register-based suicidal behavior in the Swedish workforce (22).

We found that workplace bullying was a statistically significant risk factor for suicidal behavior among men. This is in accord with a few previous studies suggesting that the association between workplace bullying and mental health is stronger among men, although the current evidence about sex-related differences remains inconclusive (23). A possible explanation for this finding is that men may tend to make less frequent use of health services (eg, professional psychological support) when confronted with adverse life circumstances (24). For instance, previous research found that masculinity may reduce help-seeking for depression among men (25). Reduced support seeking might lead to untreated or worsened mental disorders, which may in turn increase the risk of suicidal behavior (11). In addition, men might rely on their work role to establish their self-identity more than women do, which may result in mental health being more strongly affected among men when confronted with highly stressful events – such as workplace bullying – that can severely threaten one’s self-esteem (26).

### Strengths and limitations

The strengths of our study are the prospective design and the large sample size, providing sufficient statistical power to analyze the prospective association between a low-base rate phenomenon such as workplace bullying and suicidal behavior. The study was based on a detailed protocol describing the planned analytical strategy that was published before the analyses were performed (14), as a precaution against selective reporting and post-hoc decision making. In addition, suicide attempts and death by suicide were assessed using register-based data, and unique identifiers allowed us to ensure that each individual was included only once. Finally, by adjusting for register-based history of diagnosed mental disorders and psychotropic drug prescriptions, we were able to account for the fact that individuals with previous mental disorders may be at a higher risk of both being bullied and engaging in suicidal behavior (11).

This study has also limitations. Information on history of non-treated mental disorders was not available. Non-participation and missing data in the questionnaire surveys could have been related to both the reporting of workplace bullying and subsequent suicidal behavior, which might have biased our estimates. We could not account for possible confounding factors, such as personality traits, co-occurring life- and work-related traumatic events, and exposure to other psychosocial work stressors. In particular, personality traits have been associated with both the reporting of workplace bullying and suicidal behavior (27, 28). Lack of analytical power, mainly resulting from the low base-rate prevalence of suicidal behavior, prevented us from estimating dose–response associations between exposure to workplace bullying of increasing frequency and suicidal behavior. The latter might have introduced effect underestimation since the most negative consequences on mental health were previously observed in connection with the most severe degrees of exposure (ie, frequent bullying) (8). Further, it can be argued that infrequent exposure to workplace bullying (eg, being bullied “now and then”) does not constitute a chronic stressor. That we still found an association with suicidal behavior suggests that workplace bullying might lead to extreme consequences for mental health even when the exposure is not chronic, as it is the case when individuals report being bullied on an infrequent basis. At present, however, this remains speculative, and future studies with sufficient power to perform dose–response analyses are needed to shed light onto the impact that different frequencies of exposure to workplace bullying have on suicidal behavior. While in the present study a one-time exposure to workplace bullying is associated with later suicidal behavior, we were not able to examine if variation in exposure to workplace bullying over time plays a role in the size of the association. In addition, while we adjusted and stratified the analyses by socioeconomic status, we could not examine the potential confounding role of occupation. This is a limitation because previous research suggests that both the risk of workplace bullying (29) and the risk of suicide might differ according to occupation (30). Suicidal behavior, especially suicide attempt, may be under-detected, hence possibly underestimating absolute risks reported here. As suicide is a low base-rate occurrence, we had limited statistical power to detect effects of small or moderate size in the sub-group analyses. The generalizability of our findings should be ascertained in future studies examining the association between workplace bullying and suicidal behavior in other geographical contexts.

### Implications for future research

To date, there is scarce empirical evidence about the mechanisms linking workplace bullying to suicidal behavior. Future research should address such mechanisms by examining both moderators and mediators of the association between workplace bullying and suicidal behavior. For instance, factors such as feeling defeated or humiliated have been reported as potential antecedents to suicidal behavior if not mitigated by, for example, effective coping strategies or social support (11). When it comes to potential mediators, it is plausible that mental disorders, such as depression, play a substantial role in this association (8, 11). Workplace bullying might lead to enduring feelings of entrapment and humiliation, which are associated with the development of mental disorders (31). In addition, workplace bullying may provoke a severe deterioration of self-esteem and feelings of worthlessness, which have been found in association with elevated risk of suicidal behavior (32, 33). From a pathophysiological perspective, due to its long-lasting and escalating nature, workplace bullying might represent a stressor that could lead to dysregulations of the hypothalamic-pituitary-adrenal axis and subsequent physiological changes that are linked to the development of depression (34).

### Practical implications

On a clinical level, primary care clinicians and psychiatrists should be aware that patients presenting with mental health problems related to particularly severe work-related experiences, such as workplace bullying, might be at elevated risk of suicidal behavior. Our findings suggest the need to pay special attention to men. Psychotherapy could be useful for targets suffering from mental health problems. For instance, a cognitive-behavioral in-patient psychotherapeutic approach specifically developed for the treatment of individuals exposed to workplace bullying has proven effective in improving mental health (35). On top of initiatives supporting individuals who have been already exposed to workplace bullying, there is a need to implement workplace interventions aimed at reducing work-related psychosocial factors that may increase the risk of workplace bullying (eg, role conflicts and ambiguity (36)). Workplace level interventions should also include the implementation of procedures to handle cases of workplace bullying as they occur as well as conflict management initiatives to prevent conflicts from escalating into bullying (37). From a public health perspective, the new evidence reported in this study supports efforts to strengthen national and international measures against workplace bullying.

### Concluding remarks

In conclusion, our study showed that workplace bullying is associated with subsequent suicidal behavior among individuals who had not previously been recorded with a suicide attempts, while also adjusting for history of mental disorders and psychotropic drug prescriptions. In sub-group analysis, this association was statistically significant among men but not women.

## Supplementary material

Supplementary material

## References

[1] World Health Organization (2014). Preventing suicide:a global imperative.

[2] Naghavi M (2019). Global Burden of Disease Self-Harm Collaborators Global, regional, and national burden of suicide mortality 1990 2016 systematic analysis for the Global Burden of Disease Study 2016. BMJ.

[3] Turecki G, Brent DA, Gunnell D, O'Connor RC, Oquendo MA, Pirkis J (2019). Suicide and suicide risk. Nat Rev Dis Primers.

[4] Milner A, Witt K, LaMontagne AD, Niedhammer I (2018). Psychosocial job stressors and suicidality:a meta-analysis and systematic review. Occup Environ Med.

[5] Einarsen S, Hoel H, Zapf D, Cooper C, Einarsen S, Hoel H, Zapf D, Cooper C (2020). The Concept of Bullying and Harassment at Work:The European Tradition. Bullying and harassment in the workplace:Theory, research and practice.

[6] Nielsen MB, Matthiesen SB, Einarsen S (2010). The impact of methodological moderators on prevalence rates of workplace bullying. A meta-analysis. J Occup Organ Psychol.

[7] Theorell T, Hammarström A, Aronsson G, Träskman Bendz L, Grape T, Hogstedt C (2015). A systematic review including meta-analysis of work environment and depressive symptoms. BMC Public Health.

[8] Gullander M, Hogh A, Hansen ÅM, Persson R, Rugulies R, Kolstad HA (2014). Exposure to workplace bullying and risk of depression. J Occup Environ Med.

[9] Nielsen MB, Nielsen GH, Notelaers G, Einarsen S (2015). Workplace Bullying and Suicidal Ideation:A 3-Wave Longitudinal Norwegian Study. Am J Public Health.

[10] Nielsen MB, Einarsen S, Notelaers G, Nielsen GH (2016). Does exposure to bullying behaviors at the workplace contribute to later suicidal ideation?A three-wave longitudinal study. Scand J Work Environ Health.

[11] O'Connor RC, Nock MK (2014). The psychology of suicidal behaviour. Lancet Psychiatry.

[12] Mikkelsen EG, Hansen ÅM, Persson R, Byrgesen MF, Hogh A, Einarsen S, Hoel H, Zapf D, Cooper C (2020). Individual Consequences of Being Exposed to Workplace Bullying. Bullying and harassment in the workplace:Theory, research and practice.

[13] Leach LS, Poyser C, Butterworth P (2017). Workplace bullying and the association with suicidal ideation/thoughts and behaviour:a systematic review. Occup Environ Med.

[14] Conway PM, Erlangsen A, Grynderup MB, Clausen T, Bjørner J, Burr H (2020). Study Protocol:The association between workplace bullying and suicidal behaviour. A register-based prospective study of 98,330 participants in Denmark.

[15] Erlangsen A, Fedyszyn I (2015). Danish nationwide registers for public health and health-related research. Scand J Public Health.

[16] Pedersen CB (2011). The Danish Civil Registration System. Scand J Public Health.

[17] Lynge E, Sandegaard JL, Rebolj M (2011). The Danish National Patient Register. Scand J Public Health.

[18] Mors O, Perto GP, Mortensen PB (2011). The Danish Psychiatric Central Research Register. Scand J Public Health.

[19] Helweg-Larsen K (2011). The Danish Register of Causes of Death. Scand J Public Health.

[20] Kildemoes HW, Sørensen HT, Hallas J (2011). The Danish National Prescription Registry. Scand J Public Health.

[21] Williams RL (2000). A note on robust variance estimation for cluster-correlated data. Biometrics.

[22] Magnusson Hanson LL, Nyberg A, Mittendorfer-Rutz E, Bondestam F, Madsen IE (2020). Work related sexual harassment and risk of suicide and suicide attempts:prospective cohort study. BMJ.

[23] Boudrias V, Trépanier SG, Salin D (2021). A systematic review of research on the longitudinal consequences of workplace bullying and the mechanisms involved. Aggress Violent Behav.

[24] Juvrud J, Rennels JL (2017). “I Don't Need Help”:Gender Differences in how Gender Stereotypes Predict Help-Seeking. Sex Roles.

[25] Seidler ZE, Dawes AJ, Rice SM, Oliffe JL, Dhillon HM (2016). The role of masculinity in men's help-seeking for depression:A systematic review. Clin Psychol Rev.

[26] Wiley MG (1991). Gender, Work, and Stress:The Potential Impact of Role-Identity Salience and Commitment. Sociol Q.

[27] Nielsen MB, Knardahl S (2015). Is workplace bullying related to the personality traits of victims?A two-year prospective study. Work Stress.

[28] Brezo J, Paris J, Turecki G (2006). Personality traits as correlates of suicidal ideation, suicide attempts, and suicide completions:a systematic review. Acta Psychiatr Scand.

[29] Einarsen S, Hoel H, Zapf D, Cooper C, Einarsen S, Hoel H, Zapf D, Cooper C (2020). Empirical Findings on Prevalence and Risk Groups of Bullying in the Workplace. Bullying and harassment in the workplace:Theory, research and practice.

[30] Milner A, Spittal MJ, Pirkis J, LaMontagne AD (2013). Suicide by occupation:systematic review and meta-analysis. Br J Psychiatry.

[31] Kendler KS, Hettema JM, Butera F, Gardner CO, Prescott CA (2003). Life event dimensions of loss, humiliation, entrapment, and danger in the prediction of onsets of major depression and generalized anxiety. Arch Gen Psychiatry.

[32] Bi B, Xiao X, Zhang H, Gao J, Tao M, Niu H (2012). A comparison of the clinical characteristics of women with recurrent major depression with and without suicidal symptomatology. Psychol Med.

[33] Calati R, Ferrari C, Brittner M, Oasi O, Olié E, Carvalho AF (2019). Suicidal thoughts and behaviors and social isolation:A narrative review of the literature. J Affect Disord.

[34] Gold PW (2015). The organization of the stress system and its dysregulation in depressive illness. Mol Psychiatry.

[35] Schwickerath J, Zapf D, Einarsen S, Hoel H, Zapf D, Cooper C (2020). Inpatient Psychotherapy of Bullying Victims. Bullying and harassment in the workplace:Theory, research and practice.

[36] Reknes I, Einarsen S, Knardahl S, Lau B (2014). The prospective relationship between role stressors and new cases of self-reported workplace bullying. Scand J Psychol.

[37] Zapf D, Vartia M, Einarsen S, Hoel H, Zapf D, Cooper C (2020). Prevention and Treatment of workplace bullying. Bullying and harassment in the workplace:Theory, research and practice.

