# Supplementary material for: Workplace bullying and risk of suicide and suicide attempts: A register-based prospective cohort study of 98 330 participants in Denmark
Source: Scand J Work Environ Health. 2022 Aug 31;48(6):425–34. doi: 10.5271/sjweh.4034 (PMC9888442; doi:10.5271/sjweh.4034)
Supplement: Supplementary material [file SJWEH-48-425-S001.pdf]

Workplace bullying and risk of suicide and suicide attempts: A register-based prospective cohort study of 98 330 participants in Denmark[1]

by Paul Maurice Conway, PhD,[2] Annette Erlangsen, PhD, Matias Brødsgaard Grynderup, PhD, Thomas Clausen, PhD, Reiner Rugulies, PhD, Jakob Bue Bjorner, PhD, Hermann Burr, PhD, Laura Francioli, PhD, Anne Helene Garde, PhD, Åse Marie Hansen, PhD, Linda Magnusson Hanson, PhD, Jonas Kirchheiner-Rasmussen, MSc, Tage S Kristensen, DrMedSci, Eva Gemzøe Mikkelsen, PhD, Elsebeth Stenager, MD, Sannie Vester Thorsen, PhD, Ebbe Villadsen, BSc, Annie Høgh, PhD

1. Supplementary material
2. Correspondence to: Paul Maurice Conway, Department of Psychology, University of Copenhagen, Øster Farimagsgade 2A, 1353 Copenhagen, Denmark. [E-mail: paul.conway@psy.ku.dk].

Supplementary table S1. Surveys included in the pooled dataset.

	Description
**Survey**	
DWECS 2005	The Danish Work Environment Cohort Study 2005. National, representative sample of employed persons conducted in 2005.
DWECS 2010	The Danish Work Environment Cohort Study 2005. National, representative sample of employed persons conducted in 2010.
COPSOQ II	Copenhagen Psychosocial Questionnaire Study II. National, representative sample of employed persons conducted in 2004.
PRISME 2007	Psychosocial RIsk factors for Stress and MEntal disease cohort. Workplace-based survey in public workplaces in the Regional sector in Denmark conducted in 2007.
SATH	Nursing Work Environment, Well-being and Health. National, representative sample of registered nurses in Denmark conducted in 2007.
WBH 2006	Workplace Bullying and Harassment cohort 2006. Workplace-based survey in private and public workplaces in Denmark conducted in 2006.
WBH 2008	Workplace Bullying and Harassment cohort 2008. Workplace-based survey in private and public workplaces in Denmark conducted in 2008.
SOSU I	Social and Health Care Study I. Workplace-based survey among employees in the Danish Eldercare services conducted in 2004/2005.
SOSU II	Social and Health Care Study II. Workplace-based survey among employees in the Danish Eldercare services conducted in 2006/2007.
SOSU III	Social and Health Care Study III. Workplace-based survey among employees in the Danish Eldercare services conducted in conducted in 2008/2009.
SOSU U	The Danish Health Care Worker Cohort-Class of 2004. Survey among all newly educated social and health care helpers and assistants conducted in 2004.
DANES 2008	Danish National Working Environment Study (DANES): A national, representative sample of employed persons conducted in 2007.
WEHD 2012	Work Environment and Health 2012. National, representative sample of employed persons conducted in 2012.
WEHD 2014	Work Environment and Health 2014. National, representative sample of employed persons conducted in 2014.

Supplementary table S2. Characteristics of study sample (n=98 330).

	DWECS 2005	DWECS 2010	COPSOQ II	PRISME 2007	SATH	WBH 2006	WBH 2008	SOSU I	SOSU II	SOSU III	SOSU U	DANES 2008	WEHD 2012	WEHD 2014
	N (%)													
**Sample**	8878 (9.0)	5997 (6.1)	3388 (3.5)	4364 (4.4)	4543 (4.6)	3172 (3.2)	564 (0.6)	9631 (9.8)	3537 (3.6)	2414 (2.5)	2362 (2.4)	10 410 (10.6)	23 053 (23.4)	16 017 (16.3)
**Age mean (SD)**	42.5 (11.4)	41.1 (11.9)	42.3 (10.2)	44.6 (10.2)	44.2 (9.9)	44.9 (10.1)	40.8 (10.5)	45.5 (10.0)	42.7 (10.9)	41.9 (11.6)	36.5 (10.7)	46.6 (11.4)	45.5 (11.2)	45.8 (11.3)
**Age dichotomized**														
**<31**	1513 (7.0)	1329 (2.2)	513 (15.1)	489 (1.2)	519 (1.4)	280 (8.8)	89 (5.8)	899 (9.3)	605 (7.1)	493 (0.4)	803 (4.0)	1164 (1.2)	2715 (11.8)	1916 (12.0)
**≥31**	7365 (3.0)	4668 (7.8)	2875 (84.9)	3875 (8.8)	4024 (8.6)	2892 (1.2)	475 (4.2)	8732 (0.7)	2932 (2.9)	1921 (9.6)	1559 (6.0)	9246 (8.8)	20 338 (88.2)	14 101 (88.0)
**Women**	4498 (0.7)	3169 (2.8)	1771 (52.2)	3421 (8.4)	4399 (6.8)	2114 (6.6)	377 (6.8)	9214 (5.7)	3304 (3.4)	2250 (3.2)	2254 (5.4)	5019 (8.2)	12294 (53.3)	8498 (53.1)
**Participants reporting exposure to workplace bullying**	810 (9.1)	632 (10.5)	281 (8.3)	359 (8.2)	460 (10.1)	339 (10.7)	51 (9.0)	1143 (11.9)	302 (8.5)	228 (9.4)	228 (9.7)	714 (6.9)	2834 (12.3)	1878 (11.7)
**Marital status**														
**Living alone**	1231 (3.9)	997 (6.6)	479 (14.1)	579 (3.3)	389 (8.6)	381 (12.0)	74 (3.1)	829 (8.6)	372 (0.5)	342 (4.2)	420 (7.8)	1271 (2.2)	2959 (12.8)	2009 (12.5)
**Cohabiting**	7104 (80.0)	4683 (78.1)	2693 (79.5)	3424 (78.5)	3784 (83.3)	2542 (80.1)	457 (81.0)	7811 (81.1)	2827 (79.9)	1861 (77.1)	1763 (74.6)	8371 (80.4)	18467 (80.1)	12781 (79.8)
**Divorced**	470 (5.3)	277 (4.5)	185 (5.5)	312 (7.2)	305 (6.7)	214 (6.8)	28 (5.0)	780 (8.1)	292 (8.3)	170 (7.0)	156 (6.6)	632 (6.1)	1350 (5.9)	1037 (6.5)
**Widow**	73 (0.8)	40 (0.7)	31 (0.9)	49 (1.1)	65 (1.4)	35 (1.1)	5 (0.9)	211 (2.2)	46 (1.3)	41 (1.7)	23 (1.0)	136 (1.3)	277 (1.2)	190 (1.2)
**Socio-economic status[a]**														
**Low**	2961 (33.4)	1880 (31.4)	1314 (38.8)	599 (13.7)	9 (0.2)	818 (25.8)	177 (31.4)	6598 (68.5)	2388 (67.5)	1716 (71.1)	2156 (91.3)	3940 (37.9)	7934 (34.4)	5105 (31.9)
**Medium**	1731 (9.5)	1159 (9.3)	731 (21.6)	2293 (2.5)	3643 (80.2)	1041 (32.8)	141 (25.0)	1502 (15.6)	609 (17.2)	335 (13.9)	<40[b] (<2.0)	2391 (23.0)	5432 (23.6)	3854 (24.1)
**High**	1668 (18.8)	1339 (22.3)	620 (18.3)	1169 (26.8)	800 (17.6)	804 (25.4)	159 (28.2)	459 (4.8)	148 (4.2)	118 (4.9)	<10[b] (<0.5)	2094 (20.1)	5588 (24.2)	4240 (26.5)
**Student/Other**	2518 (28.4)	1619 (27.0)	723 (21.3)	303 (6.9)	91 (2.0)	509 (16.1)	87 (15.4)	1072 (11.1)	392 (11.1)	245 (10.2)	166 (7.0)	1985 (19.1)	4099 (17.8)	2818 (17.6)
**Diagnoses of any mental disorder**	209 (2.4)	200 (3.3)	71 (2.1)	124 (2.8)	109 (2.4)	78 (2.5)	25 (4.4)	256 (2.7)	131 (3.7)	133 (5.5)	110 (4.7)	270 (2.6)	775 (3.4)	573 (3.6)
**Psychotropic drug prescriptions**	1335 (15.0)	1186 (19.8)	459 (13.6)	915 (21.0)	844 (18.6)	554 (17.5)	117 (20.7)	2011 (20.9)	848 (24.0)	752 (31.2)	547 (23.2)	2213 (21.3)	5290 (23.0)	3830 (23.9)

[a]For socio-economic status: "low" includes participants employed in a job requiring skills on a basic level; "medium" includes participants employed in a job requiring skills on the mid-level; "high" includes leaders, both employed and self-employed with subordinates, and participants employed in a job requiring skills on the highest level; "student/other" includes students and self-employed without subordinates.

[b]Given the small numbers, for reasons of data protection the exact number of participants with high or medium socio-economic status in the SOSU U survey was not reported.

Supplementary table S3. Comparison of sample characteristics between participants with complete and incomplete data.

	**Participants excluded due to missing data**	**Participants excluded due to missing data**[a]	**Participants in the study sample**
Sample, no.	6192	6037	98 330
Age, mean (SD)	36.9 (14.2)	36.9 (14.2)	44.5 (11.2)
Age dichotomized			
<31, no. (%)	2585 (41.7)	2524 (41.8)	13 327 (13.6)
≥31, no. (%)	3610 (58.3)	3513 (58.2)	85 003 (86.4)
Women, no. (%)	4510 (74.4)	4382 (74.2)	62 582 (63.6)
Participants who answered the question on workplace bullying (among excluded due to missing data), no.	245	244	
Participants reporting exposure to workplace bullying, among those who answered the question on workplace bullying, no. (%)	28 (11.4)	28 (11.5)	10 259 (10.4)
Participants with information on marital status (among excluded due to missing data), no.	5887	5735	
Living alone, no. (%)	1353 (23.0)	1320 (23.0)	12 332 (12.5)
Cohabiting, no. (%)	4086 (69.4)	3990 (69.6)	78 568 (79.9)
Divorced, no. (%)	388 (6.6)	366 (6.4)	6208 (6.3)
Widow, no. (%)	60 (1.0)	59 (1.0)	1222 (1.2)
Participants with information on socio-economic status (among excluded due to missing data), no.	6044	5890	
Low, no. (%)	2717 (45.0)	2604 (44.8)	37 595 (38.2)
Medium, no. (%)	376 (6.2)	373 (6.3)	≈24900[b] (≈25)
High, no. (%)	272 (4.5)	272 (4.6)	≈19210[b] (≈20)
Student/Other, no. (%)	2679 (44.3)	2604 (44.2)	16 627 (16.9)
Diagnoses of any mental disorder, no. (%)	478 (7.7)	404 (6.7)	3064 (3.1)
Psychotropic drug prescriptions, no. (%)	1455 (23.5)	1374 (22.8)	20 901 (21.3)
Suicidal behavior, no.	65	48	186
Suicide attempt, no.	56	42	150
Death by suicide, no.	9	6	41

[a] Participants with previous suicide attempts excluded.

[b] Given the small numbers, for reasons of data protection the exact number of participants with high or medium socio-economic status in the SOSU U survey was not reported. The exact total number of participants is also not reported for high and medium socio-economic status to avoid being able to calculate the numbers for SOSU U by subtraction. The symbol ≈ indicates approximate numbers.

Supplementary table S4. Hazard ratios of suicidal behavior, suicide attempt and death by suicide in the sample wherein four respondents registered with both suicide attempts and death by suicide were excluded from the analyses (n=98,326)

	**No. (%) of suicidal events**	**Person-years**	**Rate per 100 000 person-years**	**Crude HR (95% CI)**	**Model 1[a] HR (95% CI)**	**Model 2[b] HR (95% CI)**
Suicidal behavior						
Non-exposed to WB	151 (83.9)	642 556	23.50	1.00	1.00	1.00
Exposed to WB	29 (16.1)	71 220	40.71	1.73 (1.16-2.58)	1.67 (1.12-2.50)	1.56 (1.02-2.39)
Suicide attempt						
Non-exposed to WB	122 (84.1)	642 556	18.99	1.00	1.00	1.00
Exposed to WB	23 (15.9)	71 220	32.29	1.70 (1.17-2.47)	1.65 (1.14-2.39)	1.54 (1.04-2.28)
Death by suicide						
Non-exposed to WB	29 (82.9)	643 166	4.51	1.00	1.00	1.00
Exposed to WB	6 (17.1)	71 375	8.41	1.87 (0.81-4.35)	1.75 (0.71-4.28)	1.65 (0.67-4.03)

Note: HR: Hazard Ratios; WB: workplace bullying

[a]Model 1 was adjusted for sex, age, marital status, and socio-economic status.

[b]Model 2 was adjusted for the same covariates as in Model 1 plus previous history of diagnosed mental disorders.

Supplementary table S5. Hazard ratios of suicidal behavior adjusted for previous history of psychotropic drug prescriptions.

	No. (%) of suicidal events	Person-years	Rate per 100 000 person-years	Crude HR (95% CI)	Model 1[a] HR (95% CI)	Model 2[b] HR (95% CI)
Not exposed to WB	154 (82.8)	642 572	23.97	1.00	1.00	1.00
Exposed to WB	32 (17.2)	71 237	44.92	1.83 (1.20-2.78)	1.77 (1.15-2.70)	1.59 (1.01-2.48)

Note: HR: Hazard ratios; WB: workplace bullying

[a]Model 1 adjusted for sex, age, marital status, and socio-economic status.

[b]Model 2 adjusted for Model 1 covariates plus previous history of psychotropic drug prescriptions.

Supplementary table S6. Hazard ratios of suicidal behavior, stratified by follow-up: 0 to 4 years

	No. (%) of suicidal events[a]	Person-years	Rate per 100 000 person-years	Crude HR (95% CI)	Model 1[b] HR (95% CI)	Model 2[c] HR (95% CI)
Suicidal behavior						
Non-exposed to WB	87 (81.3)	331 063	26.28	1.00	1.00	1.00
Exposed to WB	20 (18.7)	38 205	52.35	1.99 (1.34-2.96)	1.90 (1.27-2.84)	1.73 (1.12-2.67)

Note: HR: Hazard Ratios; WB: workplace bullying

[a]Four participants first attempted suicide, followed by death by suicide during follow-up.

[b]Model 1 was adjusted for sex, age, marital status, and socio-economic status.

[c]Model 2 was adjusted for the same covariates as in Model 1 plus previous history of diagnosed mental disorders.

Supplementary table S7. Hazard ratios of suicidal behavior, stratified by follow-up: More than 4 years

	No. (%) of suicidal events[a]	Person-years	Rate per 100 000 person-years	Crude HR (95% CI)	Model 1[b] HR (95% CI)	Model 2[c] HR (95% CI)
Suicidal behavior						
Non-exposed to WB	66 (85.7)	311 508	21.19	1.00	1.00	1.00
Exposed to WB	11 (14.3)	33 021	33.31	1.58 (0.73-3.47)	1.57 (0.96-3.43)	1.51 (0.69-3.30)

Note: HR: Hazard Ratios; WB: workplace bullying

[a]Four participants first attempted suicide, followed by death by suicide during follow-up.

[b]Model 1 was adjusted for sex, age, marital status, and socio-economic status.

[c]Model 2 was adjusted for the same covariates as in Model 1 plus previous history of diagnosed mental disorders.

Supplementary table S8. Hazard ratios of suicidal behavior, suicide attempt and death by suicide with full results for covariates.

	**Crude HR (95% CI)**	**Model 1[a] HR (95% CI)**	**Model 2[b] HR (95% CI)**
Suicidal behavior			
Non-exposed to WB	1.00	1.00	1.00
Exposed to WB	1.83 (1.20-2.78)	1.77 (1.15-2.70)	1.65 (1.06-2.58)
Sex (male)		1.18 (0.98-1.42)	1.24 (1.02-1.50)
Age		0.98 (0.97-0.99)	0.98 (0.97-0.99)
Marital status			
Unmarried (Reference)		1.00	1.00
Married		1.14 (0.86-1.51)	1.22 (0.92-1.63)
Divorced		2.00 (1.11-3.61)	1.88 (1.04-3.38)
Socio-economic status			
High (Reference)		1.00	1.00
Medium		2.86 (1.39-5.88)	2.85 (1.38-5.86)
Low		3.75 (1.77-7.95)	3.70 (1.76-7.78)
Other		4.20 (2.18-8.06)	3.98 (2.10-7.54)
Previous history of diagnosed mental disorders			5.21 (3.16-8.59)
Suicide attempt			
Non-exposed to WB	1.00	1.00	1.00
Exposed to WB	1.81 (1.22-2.70)	1.77 (1.19-2.63)	1.65 (1.09-2.50)
Sex (male)		0.91 (0.72-1.15)	0.95 (0.75-1.21)
Age		0.98 (0.96-0.99)	0.98 (0.97-0.99)
Marital status			
Unmarried (Reference)		1.00	1.00
Married		1.28 (0.85-1.94)	1.38 (0.91-2.09)
Divorced		1.75 (0.85-3.61)	1.64 (0.80-3.39)
Socio-economic status			
High (Reference)		1.00	1.00
Medium		3.10 (1.36-7.10)	3.09 (1.35-7.11)
Low		4.29 (1.83-10.08)	4.23 (1.18-9.89)
Other		4.74 (2.28-9.86)	4.50 (2.20-9.22)
Previous history of diagnosed mental disorders			5.31 (3.09-9.13)
Death by suicide			
Non-exposed to WB	1.00	1.00	1.00
Exposed to WB	2.34 (0.98-5.62)	2.21 (0.87-5.64)	2.08 (0.82-5.27)
Sex (male)		3.17 (1.48-6.78)	3.31 (1.49-7.35)
Age		0.99 (0.97-1.01)	0.99 (0.97-1.02)
Marital status			
Unmarried (Reference)		1.00	1.00
Married		0.79 (0.34-1.81)	0.85 (0.34-2.11)
Divorced		2.55 (0.68-9.55)	2.40 (0.67-8.60)
Socio-economic status			
High (Reference)		1.00	1.00
Medium		2.61 (0.72-9.49)	2.60 (0.72-9.34)
Low		2.57 (0.80-8.26)	2.54 (0.79-8.15)
Other		3.52 (1.38-8.99)	3.30 (1.29-8.43)
Previous history of diagnosed mental disorders			5.25 (1.69-16.28)

Note: HR: Hazard Ratios; WB: workplace bullying; Results are not reported for widowed respondents due to too few cases of suicidal events.

[a]Model 1 was adjusted for sex, age, marital status, and socio-economic status.

[b]Model 2 was adjusted for the same covariates as in Model 1 plus previous history of diagnosed mental disorders.

Supplementary figure 1. Kaplan-Meier curve for the probabilities of suicide attempt for participants exposed (solid line) and not exposed (dashed line) to workplace bullying.

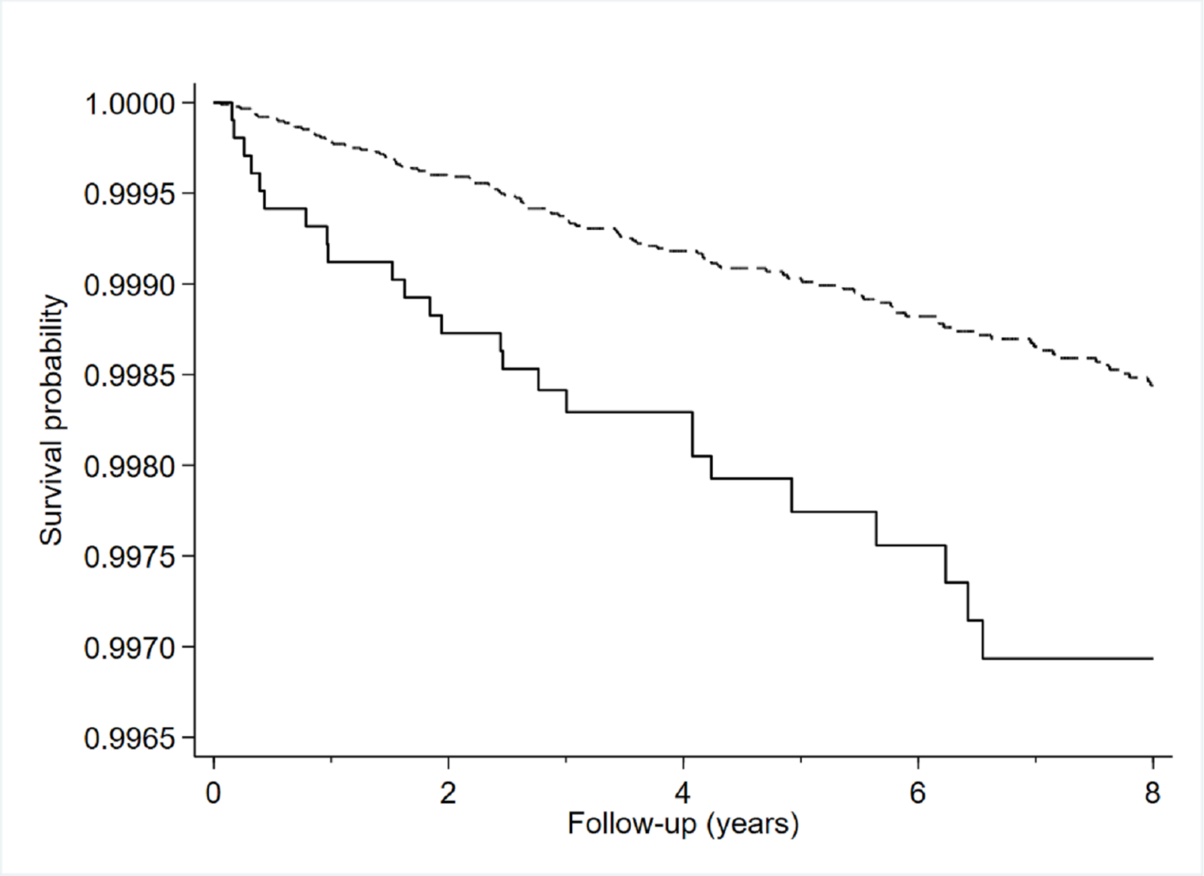

No. at risk

Exposed	10 259	10 160	8204	5348	4638
Non-exposed	88 071	87 337	72 712	51 993	45 916

Supplementary figure 2. Kaplan-Meier curve for the probabilities of death by suicide for participants exposed (solid line) and not exposed (dashed line) to workplace bullying.

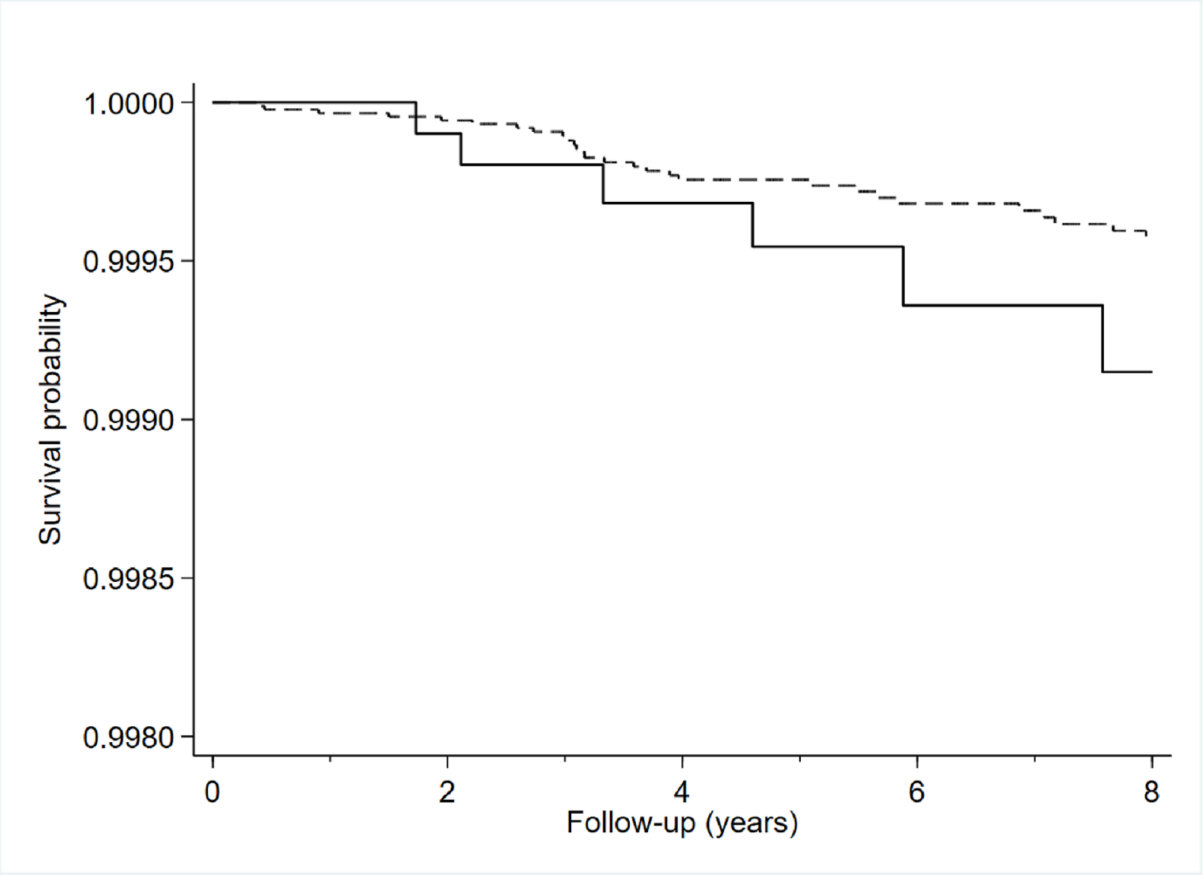

No. at risk					
Exposed	10 259	10173	8220	5365	4657
Non-exposed	88 071	87 372	72 778	52 064	45 996
